# Synergistic effects of nano SnO_2_ and TiO_2_ on the mechanical and antibacterial properties of HDPE

**DOI:** 10.1038/s41598-026-37745-y

**Published:** 2026-02-21

**Authors:** Eslam Syala, Abdallah S. Elgharbawy, Salah F. Abdellah Ali, R. A. Elsad

**Affiliations:** 1 Department of Materials Science, Institute of Graduate Studies and Researches (IGSR), Alexandria University, 163 Horreya Avenue, Shatby, 21526 Alexandria, Egypt; 2 Chemistry Department, College of Science, Al-Jouf University, 2014, Sakaka, Saudi Arabia; 3 Basic Engineering Science Department, Faculty of Engineering, Menoufia University, 32511 Shebin El Kom, Egypt

**Keywords:** High-density polyethylene (HDPE); nano-additives, nano-SnO_2_, nano-TiO_2_, Chemistry, Engineering, Materials science, Nanoscience and technology

## Abstract

This research dealt with the synthesis of both nano stannic oxide (SnO₂) and titanium dioxide (TiO₂) nanoparticles and how their addition to high-density polyethylene (HDPE) with 5 and 3%, respectively, can affect its properties from structural, mechanical, and permeability properties, besides the antibacterial activity. The possible modulation of HDPE plastic-elastic mechanical behavior was explored for each nanoparticle type and concentration. Adding 3% SnO₂ to HDPE notably improved mechanical properties in terms of toughness from 12.1 to 16.6 × 10^6^ (J/m^3^), fracture strength from 1.76 to 15.29 (MPa), and impact strength from 61.1 to 69.2 (J/m^2^), besides increasing the ductility of HDPE via decreasing the Young’s modulus from 1.75 to 1.62 (GPa). This may be ascribed to the homogeneous distributions of nano-sized inorganic fillers. This made the HDPE have unique superior properties, making it suited for applications that need engineering designs. Similarly, adding a little amount of TiO₂ (1 wt%) improved attributes like toughness to 12.30 × 10^6^ (J/m^3^) and fracture strength to 11.29 (MPa), impact strength to 63.1 (J/m^2^). The observed changes in the tested mechanical properties were influenced by filler dispersion, interfacial interaction between matrix and nano-filler, and matrix restriction mechanisms. On the contrary, high TiO_2_ filler loading (3% wt.) resulted in agglomeration and interfacial structural defects, leading to deterioration of the HDPE’s mechanical properties. The enhanced properties of the HDPE-nano SnO₂ composites encouraged the production of films of these compositions to study their water vapor and Oxygen permeabilities, besides the antibacterial activity. Increasing nano SnO_2_ percentage in HDPE matrix enhanced antibacterial functioning against both E. coli and MRSA, as mirrored by larger inhibition zones and lower obtained MIC values.

## Introduction

Nanotechnology is an emerging multidisciplinary technology that has seen fast growth in a number of domains over the previous few decades, including materials science, mechanics, polymers, and a wide range of applications^1,2^. The core idea behind nanotechnology is changing the substances’ characteristics substantially when their sizes are lowered to the nanoscale area. When material is fragmented into minute parts having one or more dimensions in the nano range, the lone particles display unanticipated characteristics and behaviors that notably differ from bulk materials^3,4^. The primary role of nanoparticles is to increase mechanical strength while also improving physical qualities, including thermal stability, conductivity, and antistatic behavior. These nanoparticles offer greater chances for interaction with polymer matrices due to their wide surface areas. They may occupy the vacancies between the chains, restricting their movement, reducing their mobility, and thus increasing the polymer’s toughness and abrasion resistance. Furthermore, nanoparticles can transfer stress away from the polymer matrix, increasing the tensile strength of nanoparticles-doped polymers^5^. One of the important characteristics leading to good-quality nanostructures, which can bring additional physical and chemical properties, is the dispersion of nano-sized additives within polymer matrices that can be achieved employing mechanical and chemical techniques^6^. Although certain nanoparticles (such as metal oxides, clays, and carbon black) have been used as micro-fillers in materials for decades, reducing them to nanoscale levels has enhanced the overall performance and sparked new economic interest^1,7^. Generally, polymer composites are composed of a polymer network and another material distributed inside it with a specified phase boundary^8^. As a result, both the macromolecular matrix and additives affect the thermal properties of polymer composites^9^. Polymer blending is a low-cost method for creating unique compounds with desirable features, resulting in objects with improved specifications and price efficiency. Tanniru^10^ discovered that adding calcium carbonate to HDPE enhanced its bulk crystallinity and modulus, as well as its impact strength at temperatures ranging from − 40 to 70 °C. Komárková et al.^11^ studied the use of oxide as a catalytic agent for thermos oxidative degradation of PMMA/TiO₂ nanocomposites. The composite samples were analyzed using TGA, DSC, TGA-MS, Py-GC-MS, and TEM. PMMA/TiO₂ composites with various TiO₂ loadings (5, 10, 15, and 20% wt.) were created by melt mixing. Adding 5% TiO₂ nanoparticles to PMMA enhanced thermal stability by over 400 °C, as per TGA and activation energy measurements. Py-GC-MS results revealed that TiO₂ enhanced the formation of methanol, methacrylic acid, and propanoic acid methyl ester during PMMA breakdown. This catalytic activity might be clarified by the interaction of the surface-located hydroxyl groups on the oxide particles’ surfaces with the methoxide group of the methacrylate function. Mallakpour et al. found that chiral polyesterimide PEI/TiO₂ nanocomposites, made from L-methionine and L-tyrosine amino acids, were physiologically active^12^. A sonochemical technique was employed to synthesize PEI/titanium bio-nanocomposites (BNCs), which hastened hydrolysis, increased the likelihood of a reactive system collision, and improved nanoparticle dispersion within the matrix. TEM images revealed no substantial particle agglomeration. TiO₂ nanoparticles were found to increase the thermal stability of BNCs in the 400–800 °C temperature range as deduced from TGA data. Lin^13^ developed and researched nanoscale TiO₂ (20–50 nm size) to function as a transparent UV filter and thermomechanical material. Using the free radical initiator 2, 2-azobis-iso-butyronitrile (AIBN), styrene monomer was supplied to the modified TiO₂ to initiate copolymerization with vinyl groups in bulk media. It was found that TiO₂ aggregated after being treated with 3-trimethoxysilyl propyl methacrylate. Thermal investigations demonstrated that the nanocomposites were more thermally stable and had glass transition temperatures comparable to pure PS. UV-Vis spectroscopy revealed that these nanocomposites had superior optical properties and may function as visually transparent UV filters. The study examined how modifier concentration affects physicochemical properties of TiO₂ surfaces. In general, amorphous TiO_2_ is characterized by having a wide surface area and a high band gap; it is inexpensive, low in density, and possesses elongation and ductility. While these prior studies have explored nano-TiO_2_ in other polymers like PMMA^11^ and polystyrene^13^, studies on HDPE are sparse, predominantly micro-scale, or focused on non-oxide fillers^10^. Critically, nano-SnO₂ has received almost no attention in HDPE matrices, despite its potential for mechanical stabilization due to ductility and homogeneity at low loadings, so it can be used in hot end coatings on bottles and the ceramics industries due to its mechanical stability. The current study aims to synthesize nano-sized stannic oxide (SnO₂) and titanium dioxide (TiO₂) nanoparticles and investigate how their incorporation at different loadings into the HDPE affects its structural, mechanical, permeability, and antibacterial characteristics. The study also directs towards the elucidation of modulation mechanisms of the plastic–elastic behavior of HDPE composites as a function of nanoparticle type, and the optimum concentration that maximizes strength, toughness, and antibacterial performance while minimizing agglomeration and structural defects. Where studies of the effect of amorphous nano titanium oxide and nano tin oxide on the mechanical properties of HDPE are almost rare, this study should be added to the limited literature in the field, particularly those related to the impact of nano-sized SnO₂. The current study fills this gap by systematically investigating the synergistic effects of nano-SnO₂ (up to 5 wt%) and nano-TiO₂ (up to 3 wt%) on HDPE, revealing optimal loadings that maximize mechanical properties, reduced permeability, and antibacterial activity—unlike high TiO₂ loadings that may induce agglomeration and property deterioration. These findings advance HDPE for multipurpose utilizations, such as durable engineering films for packaging and antimicrobial surfaces for medical or food-contact materials.

## Experimental procedures (materials, methods, and characterization)

### Materials

Both titanium isopropoxide and ethanol were provided by Sigma-Aldrich. The high-density polyethylene powder, ammonia, and dehydrated stannous chloride were supplied by Chemajet Chemical Co. and Egyptian Ethylene and Derivatives Co. (Ethydco), Egypt. The standard bacterial pathogen strains, *Staphylococcus aureus* MRSA (gram-positive), and *Escherichia coli* (gram-negative) test strains were provided by Microbiology and Immunology Dep, Faculty of Medicine (Boys), Al-Azhar University.

### Methods

#### Preparation of nano oxide filler

##### Stannic oxide

To produce SnO₂ nanoparticles, 2 g (0.1 M) stannous chloride dihydrate (SnCl₂0.2 H₂O) was solvated in 100 mL of distilled water. Following the complete dissolution, a dropwise addition of ammonia solution to the previously prepared solution was added during the parallel stirring. The produced gels were filtered and dried at 80 °C for 24 h to eliminate any residual water molecules. After 2 h of heating at 550 °C, tin oxide nanoparticles were formed^14,15^.

##### Titanium dioxide

For making amorphous Titanium dioxide nano powders, 7.4 mL of tetra-titanium iso peroxide was mixed with 6 mL of distilled water and 194 mL of ethanol. After an hour of mixing with a magnetic stirrer, the ingredients formed a gel floating on the surface of the liquid. The mixture was securely covered for 36 h to ensure that all nanoparticles precipitate correctly. Finally, the mixture was kept in a furnace at 100 °C for 8 h to produce nano titanium oxide^16,17^.

#### Compounding

HDPE-based nanocomposite films were prepared via melt compounding followed by compression molding. Initially, virgin HDPE pellets were dry-blended with metal oxide nano powders—SnO₂ (1–5 wt%) and TiO₂ (1–3 wt%)—in a Robot Coupe Blixer 4 high-speed mixer, France, for 6 min at 3000 rpm. This step ensured uniform dispersion of the powders within the HDPE matrix before extrusion. The dry blend was then fed into a single-screw extruder (Allrounder 221 K, Arburg, Germany) for melt compounding. HDPE was melted at 130 °C, and mixing was performed at high speed to produce a homogeneous molten mixture. Extruder zone temperatures were set as follows: feed zone (T1) at 185 °C, T2 at 185 °C, T3 at 190 °C, T4 at 190 °C, and nozzle (T5) at 190 °C. Operating parameters to yield extruded pellets included a screw speed of 90 cm/s, injection pressure of 1300 bar, and a 3 s cooling time. Finally, the compounded pellets were compression-molded into nanocomposite sheets (150 × 150 × 3 mm) using a HEXAPLAST compression molding machine (India) at 170–180 °C under 140 bar pressure for 10 min, followed by air cooling to room temperature. These sheets constitute the HDPE films referenced throughout the study. Dumbbell-shaped tensile specimens were cut from the sheets per ASTM D638-03 using standard hollow die punches. Additionally, bar-shaped specimens (80 × 10 × 3 mm) were prepared for flexural and Izod impact tests per ASTM D256-06a.

#### HDPE-nano-SnO_2_ films forming

HDPE/SnO_2_ films were made by adding 0.5, 1.0, and 2.0% concentrations of SnO_2_ to water w/w (based on HDPE weight). These concentrations were applied following the criteria in^18^. The solutions were then heated for 1 h on a hot plate that had been previously set to 70 °C. They were then shaken for 24 h without any heating to obtain homogenous solutions under ultrasonication settings at 50 °C, 1 h. Glycerol was used as a plasticizing agent at 40% w/w (on a dry basis). The HDPE composite samples were heated and swirled on a hot plate at 90 °C for an hour. The temperature was then lowered to 40 °C. To create a film, 90 g of homogenous solution was applied to 15 × 15 cm^2^ molding plates. Films were then dried under controlled circumstances for 24 h at 40 °C and 50% relative humidity. Two types of films were produced: control film (without SnO_2_), and composite films with 0.5, 1.0, and 2.0% nano SnO_2_ as the composition indicated in Table 1. The dried films were removed from the plates and placed in a desiccator before examination^19^.

**Table 1:** The compositions of HDPE/nano SnO_2_ composite films.

Specimen code	HDPE (wt%)	Nano SnO_2_ (wt%)
S0	100	0
S1	99.5	0.5
S2	99	1.0
S3	98	2.0

### Characterization

#### Structural evaluation

The FTIR spectra of the prepared blends were obtained by PerkinElmer, Spectrum BX FTIR device, United States, applying the KBr matrix method in the 4000 –400 cm⁻¹ scanning range, with a resolution of 4 cm⁻¹. TEM imaging for the synthesized oxides was conducted using an EDX (JEOL JSM-6360LA) with sophisticated chamber vacuum technology. The crystallography of the composites was detected by using a Philips X′Pert modular powder diffractometer.

#### Mechanical testing

The tensile characteristics of the unmixed HDPE as well as its composites were tested by the universal Zwick/Z005 machine, Germany, which has a 5 kN load cell and a crosshead speed of 20 mm/min under 23 ± 2 °C temperature and 50 ± 5% humidity lab conditions. Specimens were attached to the dynamometer’s grips and stretched with a uniform strain rate of 5 mm/min till they snapped. The impact strength (polymers’ resistance to fracture) was tested using the CEAST, Germany, testing equipment following ASTM D 256-UN. Samples were adapted in the lab for 24 h before testing in the laboratory circumstances. They were carefully subjected to the stress test using a 7.5 J hammer load. Flexural strength test was performed using a 100kN Instron 3382 dual-column floor model three-point loading universal testing machine following ASTM D-790. All experiments included three duplicate samples of each mixture.

#### Morphology studying

The surface morphology of the as-prepared composites was explored employing a Joel instrument (JEOL- JSM-IT200 Series) at an accelerating voltage of 25 kV. The specimens were sputter-coated with a thin gold coating layer (by a JEOL ion sputtering apparatus (JFC-1100E)) before imaging.

#### Water vapor and oxygen permeability testing

The water vapor permeability of the produced HDPE-nano SnO_2_ films was measured following the ASTM E96/E96M-16 method. After that, the films were exposed to 100% moisture for two days, and the amount of permeability was measured at 25 °C by weighing every two hours. The oxygen permeability of the films was estimated using the ASTM D3985-17 technique.

#### Anti-bacterial assessment of HDPE -SnO_2_ composite films

The antibacterial activity was carried out against the standard bacterial pathogen strains, *Staphylococcus aureus* MRSA, as a gram-positive, and *Escherichia coli* as a gram-negative test strain. Bacterial strains were preliminarily activated using Nutrient Broth (NB, Conda Lab, Spain) culture medium at 37 °C for 24 h at oscillation setting, then 50 µL was injected into NB after sequential dilution, and the colony-forming units (CFU)/ml of the tested bacteria were determined. Accordingly, after adjusting the inoculum concentration, it was adjusted to 10^6^ /mL. The sensitivity of *Staphylococcus aureus* MRSA and *Escherichia coli* against Cephredem and Ciprofloxacin was rated to determine the resistance ratios of the examined strains. Antibacterial activities of the tested compounds were screened at a concentration of 20 µg/mL following the agar well diffusion approach using Mueller-Hinton Agar^20^. The ability of each composite to inhibit bacterial augmentation was followed according to the inhibition zone diameter (mm) compared to the standard antibacterial drugs^21^. Thereafter, the Minimum Inhibition Concentration (MIC) of the as-prepared composites was investigated related to the standard drug by the broth microdilution method^22^. Summarily, 10 mg of every composite and reference drug was dissolved in 1 ml of sterilized deionized water to make the stock solution. Ten dilutions were prepared with a tenfold concentration larger than the final solution of the synthesized composites. The solutions were then diluted to 1:5% in Mueller Hinton Broth (MHB), and 100 µl aliquots were gradually supplied into the microdilution plates to get the wanted concentrations, which range from 5 to 160 µg/ml. Each investigated composition was incubated with the tested bacterial strain for 24 h. at 37 °C. MHB merged with deionized water, functioned as the control growth samples.

The absorbance of the suspension at 630 nm was utilized to determine growth curves of each of the treated bacterial pathogens. The percentage of growth inhibition and cell survival in both cured and uncured cells, which were checked via the turbidometry mechanism, was quantified using the equation:

$$\:A - \frac{B}{A} \times 100$$

where A and B are the absorbances of the uncured and cured samples, successively. Identification of the MIC value for each composite was referred to the minimum concentration that yielded a lower number of bacterial cells compared to the untreated samples.

## Results and discussion

### Characterization of the synthesized SnO_2_ nanoparticles

**Fig. 1:**
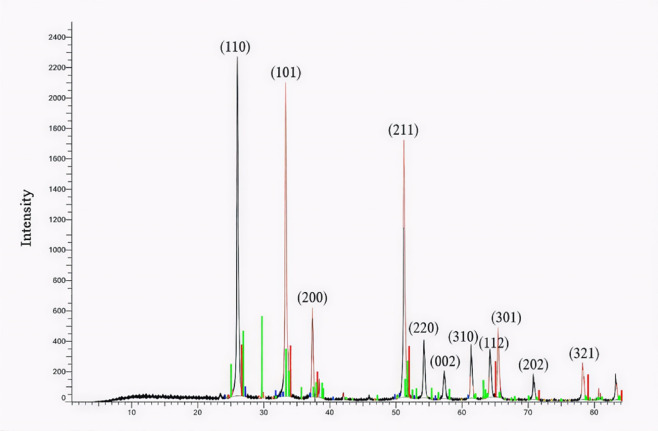
XRD spectrum of the synthesized tin oxide (SnO_2_).

**Fig. 2:**
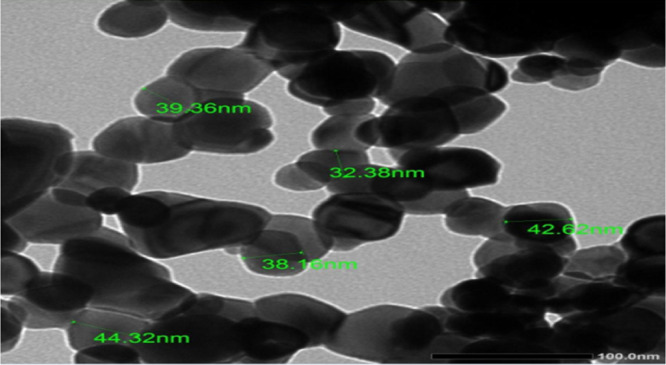
TEM illustration of the synthesized tin oxide (SnO_2_).

The XRD pattern of SnO₂ (JCPDS No. 00-001-0657) shows crisp and strong peaks that match closely, as exhibited by Fig. 1. The peaks of 26.68, 33.97, 37.95, 51.89, 54.72, 58.05, 61.94, 64.87, 66.95, 71.45, and 78.85° at 2θ agreed with the (110), (101), (200), (211), (220), (002), (310), (112), (301), (202), and (321) crystal planes, successively. The absence of any impurity peaks attests to the formation and extreme crystallinity of pure SnO₂ nanoparticles. The synthesized SnO₂ crystal structure was found to be tetragonal with (space group number: 138; lattice constants: a = 4.72 Å, b = 4.72 Å, and c = 3.17 Å). The TEM image of the synthesized SnO₂ (Fig. 2) reveals that it has a size range of 32.4–44.3 nm, which achieves its nanoscale^23^.

### Characteristics of the synthesized TiO_2_ nanoparticles

**Fig. 3:**
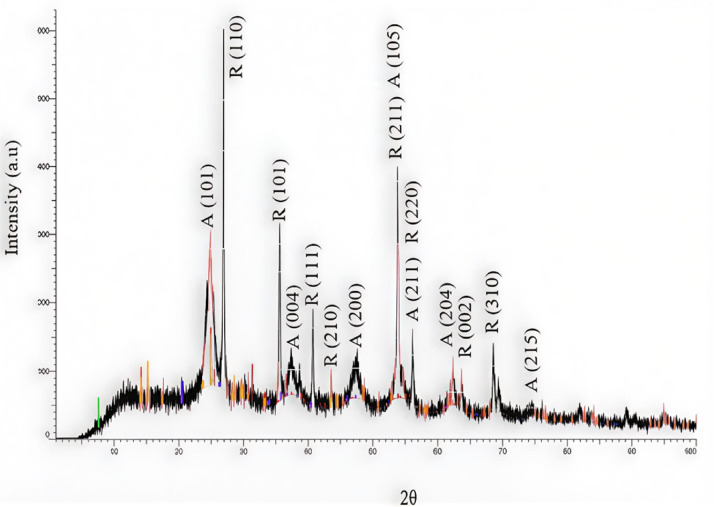
XRD spectrum of the synthesized titanium oxide (TiO_2_).

**Fig. 4:**
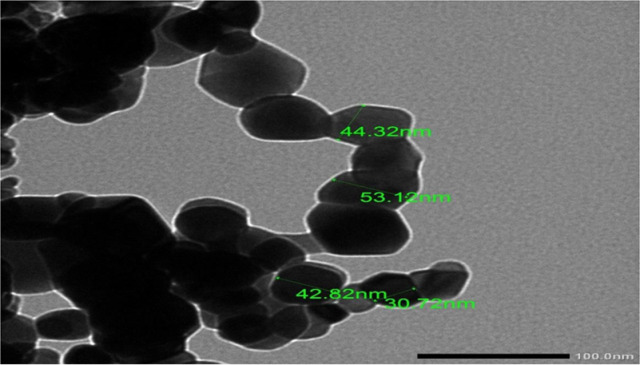
TEM image of the synthesized titanium oxide (TiO_2_).

Figure 3 reveals the XRD profile of the yielded TiO₂ nanoparticles, which indicates having a tetragonal structure, as shown by the peaks comparable to anatase and rutile in the XRD pattern. Anatase (JCPDS Card no. 21-1272) contains peaks at 2θ of 24.8, 37.3, 47.6, 53.5, 55.1, 62.2, and 74.6º, which correspond to the (101), (004), (200), (105), (211), (204), and (215) crystal planes. Rutile (JCPDS Card no. 21-1276) peaks emerge at 2θ values of 27, 35.6, 40.8, 54.0, 53.9, 56.1, and 61.0°, which are the same as crystal planes of (110), (101), (200), (111), (210), (211), (220), (002), and (310)^24^. Both of them match the profile of the formed TiO_2_ nanoparticles, which also indicate high purity and crystallinity due to the absence of impurity peaks. The synthesized TiO₂ were found to lie in the size range of 30.7–53.1 nm, as shown in their TEM image (Fig. 4), confirming their nanoscale microscopic structure.

### Identifying the chemical structure and specific functional groups of HDPE and HDPE composites

**Fig. 5:**
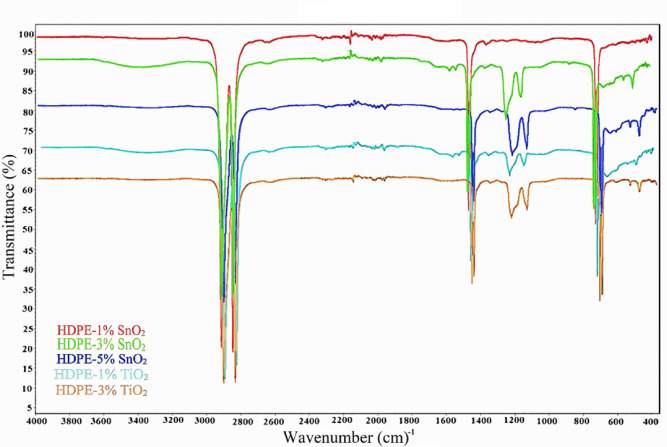
The mid-infrared spectral bands of the HDPE and HDPE-nano SnO_2_/TiO_2_ composites.

Figure 5 displays the FTIR spectra of HDPE and its composites with varying amounts of SnO₂ and TiO₂ nanoparticles. The characteristic peaks of Sn–O and Ti–O vibration (at 500–600 cm^- 1^) do not emerge in 1 wt% samples, which might be attributed to their poor concentration. Instead, they emerge with increasing metal oxide concentrations as indicated in the spectra of high concentrations. The C–H bending vibration is represented by the peaks in the range of 2846–2857 cm^- 1^ and the peak at 1472 cm^- 1^, which appear in all spectra^25^. Samples with various concentrations of metals show peaks in the range of 1245–1247 cm^- 1^ and at the position 1160 cm^- 1^. These peaks denote that the carbonyl group (C–O) is connected to the methylene group (–CH_2_). The existence of this peak confirms the combination of metal oxide with the C–H molecule. This also corresponds to the conclusions reached by other researchers^26,27^. The bands at 2913–2914 cm⁻¹ show the presence of the methylene molecule (CH₂) stretching. This band appears in the spectra of all samples, where it represents the monomer of PE. Table 2 summarizes all detected peaks in the mid-infrared spectra of the HDPE and HDPE-nano metal oxides samples.

**Table 2:** The mid-infrared spectral bands of the HDPE and HDPE-nano SnO^2^/TiO^2^ composites.

Wavenumber (cm^− 1^)	Assignment	Interpretation	The peak is attributed to
2913–2914	C–H_2_ stretching	C–H groups	HDPE
2846–2857	C–H stretching	C–H groups	HDPE
1462 − 1472	C–H bending vibrations	C–H groups	HDPE
1245–1247	C–O stretching bands	C–O group Splitting of the twisting (CH_2_)	incorporation of metal oxide with HDPE
1160	C–O stretching vibrations	C–O group	incorporation of metal oxide with HDPE
500–600	Sn–O vibration Ti–O vibration	metal oxide molecules	metal oxide

### Mechanical behavior of the HDPE composites

The experimental results were completely randomized using the average of three measurements. The results were hypothesized using the statistical analysis system (SAS) at the 0.05 level of probability (LSD_0.05_). Table 3 collects values of the mechanical properties of the prepared HDPE-nano SnO_2_/TiO_2_ composites, as well as the properties of pure HDPE.

**Table 3:** Mechanical properties of HDPE and HDPE-nano SnO_2_/TiO_2_ composites.

Sample	Maximum load (MPa)	Tensile stress at maximum load (MPa) LSD_0.05_= 1.42	Tensile strain at break % LSD_0.05_= 0.83	Young’s Modulus (GPa) LSD_0.05_= 44.21	Fracture strength (MPa) LSD_0.05_=1.11	Modulus of resilience (J/m^3^) LSD_0.05_ =2.06	Toughness ×10^6^ (J/m^3^) LCD_0.05_ = 0.02	Impact strength (J/m^2^) LSD_0.05_=0.40
HDPE	454	26.80 ^A^ (0.71)	792 ^A^ (1.0)	1.75 ^F^ (6.9)	1.76 ^A^ (0.24)	0.57 ^A^ (2.0)	12.10 ^C^ (0.01)	61.1 ^A^ (0.5)
HDPE-1% SnO_2_	394	21.40 ^D^ (0.87)	993 ^B^ (0.04)	1.21 ^E^ (9.8)	12.10 ^E^ (0.25)	0.48 ^A^ (1.0)	13.40 ^D^ (0.01)	64.5 ^E^ (0.29)
HDPE-3% SnO_2_	427	25.90 ^B^ (0.57)	1003 ^B^ (0.01)	1.62 ^A^ (57.0)	15.29 ^B^ (0.28)	0.56 ^BC^ (1.15)	16.60 ^D^ (0.02)	69.2 ^B^ (0.88)
HDPE-5% SnO_2_	412	25.00 ^C^ (0.28)	1025 ^B^ (0.07)	1.70 ^B^ (6.9)	12.94 ^D^ (0.14)	0.55 ^BC^ (1.15)	16.54 ^B^ (0.01)	77.8 ^D^ (0.76)
HDPE-1% TiO_2_	367	23.70 ^E^ (0.32)	835 ^B^ (0.51)	1.74 ^C^ (9.1)	11.49 ^E^ (0.21)	0.45 ^AB^ (1.0)	12.30 ^B^ (0.02)	63.1 ^E^ (0.29)
HDPE-3% TiO_2_	379	19.70 ^CD^ (1.44)	240 ^B^ (0.17)	1.93 ^D^ (13.5)	1.47 ^C^ (0.21)	0.38 ^D^ (0.58)	11.47 ^A^ (0.01)	56.8 ^C^ (0.5)

#### HDPE-nano SnO_2_ composites

##### Maximum load and tensile stress at maximum load

**Fig. 6:**
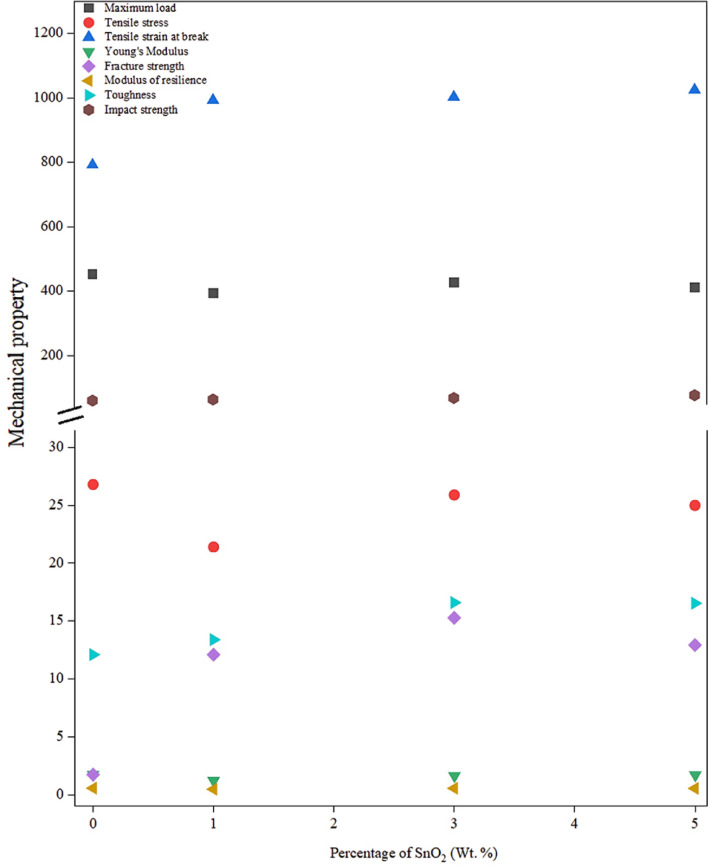
Effect of synthesized SnO_2_ nanoparticles concentration on the mechanical properties of HDPE.

Both Table 3; Fig. 6 observe the maximum load peaks at 427 MPa (25.9 MPa stress) for 3 wt% SnO₂, versus 454 MPa (26.8 MPa stress) for pure HDPE (which typically matches the HDPE literature of this section), 394 MPa (21.4 MPa stress) at 1 wt%, and 412 MPa (25 MPa stress) at 5 wt% nano SnO₂. Initial drop at 1 wt% reflects poor dispersion and stress concentrations; 3 wt% optimum yields homogeneous reinforcement via enhanced stress transfer and crystallinity, while 5 wt% decline signals agglomeration-induced defects. A similar trend was also reported for TiO₂ nanofillers-doped HDPE^28–33^.

##### Tensile strain at break, young’s modulus, and fracture strength

As displayed in both Table 3; Fig. 6, the tensile strain enhanced continuously from 729% (for neat HDPE) to 993% (at 1 wt% SnO₂), 1025% (5 wt% nano SnO₂) due to interfacial adhesion between the Sn–O surface and polyethylene chains, chain mobility restriction, and nucleation-induced crystallinity that can be induced by SnO₂ nanoparticles during HDPE crystallization^32^. This behavior further supports the presence of an optimum reinforcement threshold rather than inconsistent mechanical behavior. Young’s modulus drooped initially at 1 wt% nano SnO₂ from 1.75 to 1.21 GPa due to the induction of stress concentration, but recovered toward pure HDPE values at 3–5 SnO₂ wt% (1.62–1.70 GPa) via a continuous reinforcement network facilitating better load transfer. Additionally, the fracture strength peaks at 15.29 MPa at 3 wt% SnO₂ from 1.76 MPa for unmixed HDPE and 12.10 MPa at 1 wt% SnO_2_. This enhancement is a result of optimal bonding/load transfer between HDPE itself and SnO_2_ nano oxide particles^34^. Final declining ~ 15% at 5 wt% nano SnO₂ from 15.29 to 12.94 MPa is due to particle agglomeration^35,36^.

##### Modulus of resilience, toughness, and impact strength

**Fig. 7:**
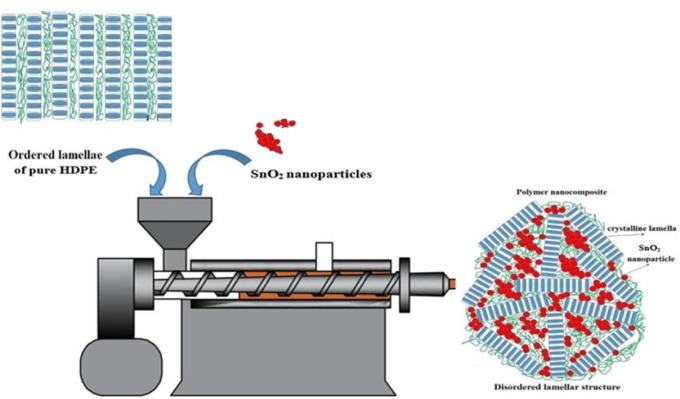
Schematic illustration of the impact of SnO_2_ nanoparticles on the HDPE lamellar structure.

Resilience fell from 0.57 (for pure HDPE) to 0.48 J/m³ (at 1 wt% SnO₂) due to weak interfaces weakening the material’s capability to absorb energy elastically^37^. After that, the resilience modulus recovered at 3–5 wt% to become (0.56–0.55 J/m^3^, respectively), as in both Table 3; Fig. 6, via improved dispersion and hence energy absorption capacity. The enhancement in resilience could also be linked to improving elastic energy storage. Toughness property increased from 12.10 (for neat HDPE) to 16.6 J/m³ (for 3 wt% SnO_2_) and 16.54 J/m³ (for 5 wt% nano SnO_2_) due to stress dissipation and crystallization, promoting the stiffness and impact resistance by restricting polymer chain mobility and crack growth pathways^38,39^. The impact strength was found to improve gradually from 61.1 (for unblended HDPE) to 77.8 J/m² (at 5 wt% nano SnO_2_), reflecting crack deflection/pinning and disrupted spherulites. The results confirm that SnO_2_ nanoparticle loading effectively reinforces the HDPE matrix for impact applications. While pure HDPE offers high thermal resistance, its 70–80% crystallinity causes stress cracking sensitivity. Compounding with SnO₂ nanoparticles enhances ductility, toughness, fracture strength, and impact strength by disordering crystalline structures—disrupting lamellae and spherulites, acting as a diluent with inter-plasticizing effects, as represented in Fig. 7^40^. Statistical superscripts in Table 3 confirm significance across loadings.

#### HDPE-nano TiO_2_ composites

##### Maximum load and tensile stress at maximum load

**Fig. 8:**
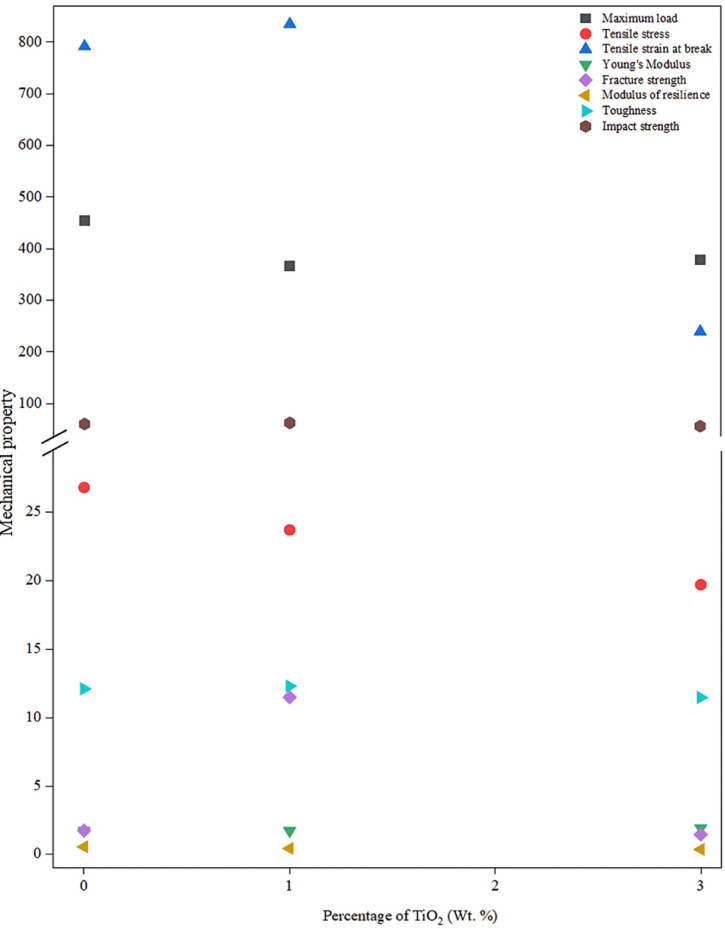
Effect of synthesized TiO_2_ nanoparticles concentration on the mechanical properties of HDPE.

**Fig. 9:**
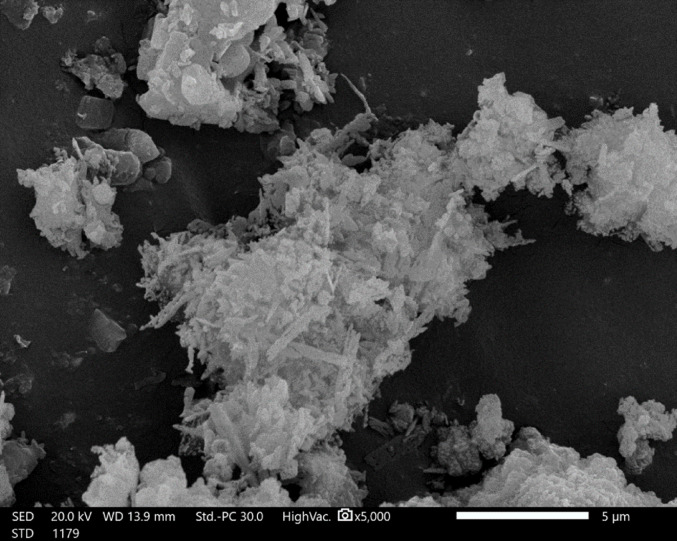
SEM image of HDPE-3% TiO_2_ composite.

Both Table 3; Fig. 8 show that pure HDPE exhibits the highest peak load (454 MPa; tensile stress 26.8 MPa). With 1% TiO₂, these values reduced to 367 MPa and 23.7 MPa. With increasing TiO₂ content to 3%, values became 379 MPa and 19.7 MPa, respectively. This decline at low-to-moderate loadings (1–3 wt%) arises from suboptimal dispersion, weak interfacial bonding, and stress concentrations acting as defects, limiting load transfer despite some nanoscale reinforcement^33,41–43^. Agglomeration at 3% exacerbates this via high surface energy clustering as seen in Fig. 9.

##### Tensile strain at break %

Table 3 indicates that the tensile strain at break increases from 792% (pure HDPE) to 835% at 1% TiO₂, then drops sharply at 3% to 240%. At 1 wt%, the good nanoparticle dispersion restricts chain mobility, enhances stress transfer, and boosts crystallinity/reinforcement without significant clustering—improving ductility. The 3% drop reflects agglomeration-induced stress concentrations and premature failure, embrittling the matrix. These results typically agree with what has been concluded by Shirkavand and Moslehifard, who found that the insertion of TiO_2_ up to 1% into the polymer matrix enhances the mechanical properties. The higher this ratio, the mechanical performance starts to deteriorate as a result of the clustering phenomenon, causing matrix embrittlement, leading to a lower tensile strain % at break^45^.

##### Young’s modulus

Young’s modulus remained stable at 1.75 GPa (pure HDPE) to 1.74 GPa (1% TiO₂), then rose to 1.93 GPa at 3%, as recorded in Table 3. Low loading has a negligible effect on the modulus, while 3% enhanced stiffness via rigid filler addition and improved stress transfer, despite agglomeration trade-offs resembling what has been reported in other nano TiO_2_-HDPE composite studies^46^. This stiffness gain occurs at the expense of ductility.

##### Fracture strength

Fracture strength surges from 1.76 MPa (neat HDPE) to 11.49 MPa at 1% TiO₂, then plummets to 1.47 MPa at 3%, as in Table 3. Optimal 1 wt% dispersion strengthens the interfacial bonding and load transfer, improving crystallinity and stress distribution, which agrees with previous studies^47^. Excess at 3 wt% causes agglomeration defects, weakening the matrix, and decreasing the needed absorbed force to fracture.

##### Modulus of resilience and toughness

Modulus of resilience decreased from 0.57 J/m³ (for pure HDPE) to 0.45 (at 1% TiO₂) and 0.38 J/m³ (at 3% TiO₂), while toughness slightly rose from 12.10 to 12.30 J/m^2^ (for 1% TiO₂) before sharper falling to 11.47 J/m³ (for 3% TiO₂) as concluded in both Table 3; Fig. 8. 1% TiO₂ aided energy dissipation via crack deflection and chain restriction^41,48^; but higher loading sacrifices toughness for stiffness due to structure embrittlement^49^. This reflects the common stiffness-toughness trade-off in nanocomposites, as reported by Mahmoud et al.^28^.

##### Impact strength

Impact strength edged up from 61.1 J/m² (for pure HDPE) to 63.1 J/m² (at 1% TiO₂), then fell to 56.8 J/m^2^ (at 3% TiO₂) as revealed by Table 3; Fig. 8. Low TiO_2_ content improved resistance by filling free volume and impeding cracks, functioning as physical crosslink points^41^; while at 3% TiO₂, the agglomeration disrupts stress uniformity, promoting brittleness. Comprehensively, hydrophilic TiO₂-hydrophobic HDPE incompatibility amplifies declines of most measured mechanical properties at high TiO₂ loadings, resulting in the observed deterioration of most measured mechanical attributes^50^. The statistical groupings in Table 3 (superscripts A, B, C, etc.) denote significant differences between composites, justifying claims regarding enhancement or reduction in the studied mechanical properties due to TiO_2_ nanofiller incorporation.

### The effect of nano SnO_2_ on the barrier features of HDPE films

**Fig. 10:**
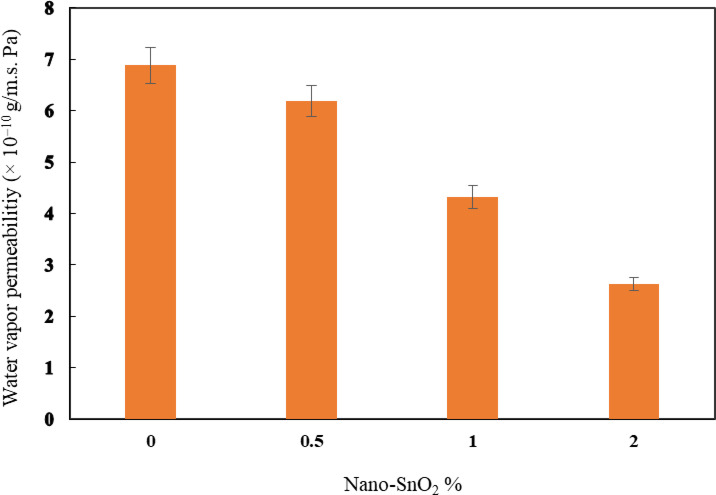
Water vapor permeability of HDPE-nano SnO_2_ films.

**Fig. 11:**
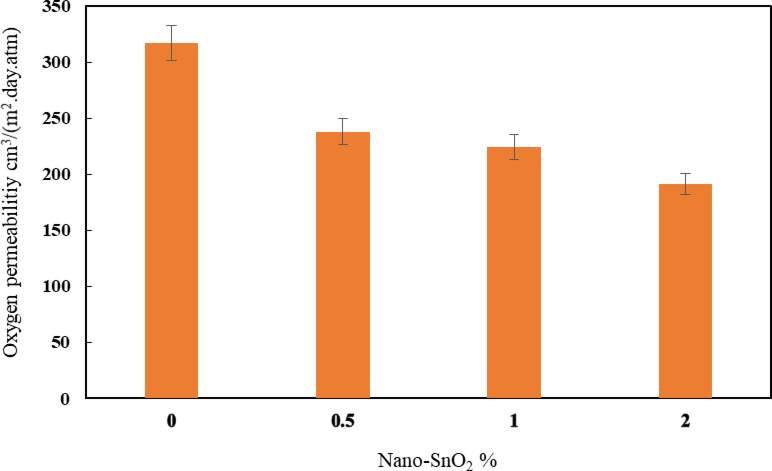
Oxygen permeability of HDPE-nano SnO_2_ nano films.

After the enhanced mechanical performance revealed by the HDPE doped with nano SnO_2_, HDPE-nano-SnO_2_ composite films were made in accordance with the composition indicated in Table 1. Figures 10 and 11 show the permeabilities of nano-films containing nano SnO₂ for water vapor and oxygen. The control sample obtained the highest values, 6.88 × 10^–10^ g/m.s. Pa and 317.02 cm^3^/(m^2^.day.atm), successively. By increasing the concentrations of nano SnO_2_ in the HDPE films network to 2%, water vapor and the permeability to oxygen significantly lowered to 2.63 × 10^–10^ (g/m.s. Pa) and 191.12 cm^3^/(m^2^.day.atm), respectively. This decrease of HDPE-based nano SnO₂-films might be attributed primarily to the fact that nano-SnO₂ behaves as an effective barrier within HDPE, likely disrupting polymer chain packing and creating a more tortuous path for water and gas molecules, which reduces their diffusion rate through the composite films compared to the neat HDPE. This integration may reduce relative gas/moisture transference, since the rapid transfer of such steams is a fundamental disadvantage of biopolymer-based films. This indicates that nano-SnO₂ effectively enhances the gas/moisture barrier properties of HDPE, which is beneficial for applications requiring enhanced packaging performance and shelf-life preservation. In general, the use of nanoparticles was discovered to significantly reduce the water vapor and oxygen permeabilities of biopolymer-based films, as detected from the literature^51^, allowing the use of these nanocomposite films in advanced packaging technologies.

### Antibacterial activities evaluation of HDPE-nano SnO_2_ composite films

The resistance of bacterial pathogens toward the classical antibacterial agent Cephradine was clearly observed, particularly for MRSA, while vancomycin exhibited only moderate activity toward MRSA compared with its effect on *E. coli*, as evidenced by the small inhibition zones and relatively high MIC values in Table 4. In contrast, the HDPE-nano SnO_2_ composites revealed a strong inhibitory effect that increased with rising SnO₂ fraction, as reflected by the larger inhibition zones and lower MIC values. Because MIC denotes the lowest concentration at which visible bacterial growth is entirely inhibited, the observed decrease in MIC with increasing SnO₂ content straightly indicates enhanced bactericidal potency of the composites^52^. As shown in Table 4, pure HDPE exhibits only moderate antibacterial activity (small inhibition zone, moderate MIC), suggesting that HDPE itself possesses limited inherent antibacterial characteristics. Among the prepared composites, S3 composition, containing 2 wt% SnO₂, reveals the largest inhibition zone and lowest MIC values against both *E. coli* and MRSA, demonstrating that this SnO_2_ level, i.e., 2%, provides the most effectual balance between nanoparticle dispersion and interfacial contact with bacterial cells. The lower MIC of S3 composite, which is equivalent to or better than that of vancomycin, emphasizes that the HDPE–SnO₂ matrix can behave as a potent antibacterial surface rather than merely a passive carrier for the nanoparticles^53^. Enhanced antibacterial effects at this level are evident for both bacterial strains. The disparity in MIC and inhibition zone trends between *E. coli* and MRSA observed in Table 4 denotes that Gram-negative and Gram-positive bacteria respond individually to the composite structure and nano SnO_2_ surface. Gram‑negative E. coli possesses an additional outer membrane that can partially hinder nanoparticle penetration and reactive oxygen species (ROS), often resulting in slightly higher MICs and smaller inhibition zones than those observed for Gram‑positive MRSA, whose thick but more porous peptidoglycan layer allows closer contact with the SnO₂ surface^54^.​ Matching preferential activity of SnO₂ and related metal‑oxide nanoparticles regarding Gram‑positive bacteria has been reported and is usually attributed to dissimilarities in cell‑wall composition, surface charge, and susceptibility to oxidative damage^55^. The observed trends can be interpreted on the basis that the incorporation of nano SnO₂ into the HDPE network betters the antibacterial activity primarily through ROS generation and direct interaction with bacterial membranes, driving lipid peroxidation, protein oxidation, and membrane disruption. Increasing the SnO₂ fraction increases the effective surface area and the number of potential contact sites for ROS production and membrane binding, which illustrates the progressive decrease in MIC and enlargement of inhibition zones up to the optimum 2 wt% SnO₂ level in S3 composite. These findings suggest a synergistic interaction between the HDPE matrix and well‑dispersed nano SnO₂ particles in suppressing bacterial growth, presenting a mechanistic basis for the improved antibacterial performance observed in Table 4^55,56^. Such composites hold potential for multifunctional applications, including synergy with cement kiln dust-based adsorbents for dyes and heavy metals-laden wastewater, thereby advancing sustainable materials that combat both microbial contamination and industrial pollution^57,58^

**Table 4:** Antibacterial activity of the prepared HDPE/nano SnO_2_ composite films using the agar well diffusion method and minimum inhibitory concentration (MIC).

Sample code	Inhibition Zone (mm)		Minimum Inhibitory Concentration (µg/mL)	
	Escherichia coli (Gram-negative)	Staphylococcus aureus MRSA (Gram-positive)	Escherichia coli (Gram-negative)	Staphylococcus aureus MRSA (Gram-positive)
S0	7	4	10	40
S1	11	5	20	10
S2	9	4	10	20
S3	12	7	5	5
Vancomycin	10	6	5	25
Cephradine	7	ND	80	ND

Vancomycin and Cephradine were used as positive control, ND: not determined.

Screening of the composites was preliminary take place at a concentration of 20 µg/mL using the agar well diffusion method.

## Conclusion

Compounding the HDPE with (1–5% wt.) nano SnO_2_ significantly enhanced the mechanical attributes of the neat matrix, including fracture strength, toughness, tensile strain at break, and impact strength attributes. This enhancement was due to the smaller weight, the large exterior area, and the compatibility of the nano additive, which promoted the stress transfer and crack-arresting effects. FTIR and microscopic studies showed that SnO_2_/TiO_2_ nanoparticles interacted physically with the HDPE network, supporting the composite reinforcement effects at low filler loading, which is dependent on the dispersion quality rather than chemical bonding. Conversely, combining TiO_2_ with the HDPE impaired the mechanical qualities of HDPE. The behavior aligns with the understanding that a small amount of TiO_2_ nanoparticles slightly reinforced the HDPE matrix by improving intermolecular interactions and absorbing impact energy. On the other side, an excess of TiO_2_ led to aggregation, weakening the composite material under overload conditions. Overall, the results reported that there was an optimum nanoparticle fraction that exists, beyond which mechanical properties deteriorate due to poor dispersion and agglomeration of filler nanoparticles. The improvements obtained in the antibacterial properties highlighted the applicability of the prepared HDPE/nano SnO_2_ composites as antimicrobial materials, particularly at higher SnO_2_ nanoparticle loadings.

## Data Availability

All data generated or analyzed during this study are included in this published article and supplementary information files.
